# Split Decisions, Split Decisions

**DOI:** 10.1100/tsw.2000.13

**Published:** 2000-11-17

## Abstract

The lead stories in Nature and Science went in opposite directions this week. Science chose outer space, launching into NASA's hotly disputed decision to shelve a planned mission to Pluto. Nature plunged into inner space with a story about a report to the European Commission advising against granting “premature” approval to create human embryos for stem-cell research.

Scientific use of stem cells from human embryos has sparked controversy around the globe, leading Germany to ban the practice. Now a new report from the 12-member European Group on Ethics in Science and New Technologies (EGE) threatens to confuse the issue even more, reports *Nature* in its lead story. The somewhat contradictory EGE report acknowledges the "scientific justification" of stem-cell research, while at the same time characterizing the hopes of regenerative medicine as "highly speculative," says *Nature*, and the EGE recommends "prudence." The bipolar tone of the report is the product of the EGE's quixotic effort to appease two factions: those who want to ban all stem-cell research and those who don't. Although the EGE claims they are "not trying to interfere in any way with national legislation," the report was released just days before the British parliament is scheduled to begin debate on regulating stem-cell research. *Science*, did not report on this story.

*Science* led with another story about a controversial political decision: NASA's postponement of a planned mission to Pluto. After a Jet Propulsion Lab (JPL) cost estimate for the mission nearly tripled from a relatively modest $650 million to a staggering $1.4 billion, NASA space science chief, Ed Weiler, said enough is enough and stopped work in September, reports *Science*. But planetary scientists think the extra cash is worth it. As astrophysicist Alan Stern says, "If Pluto waits, science will be lost." TWINS thinks he probably meant "data will be lost," but why quibble over $800 million? Some scientists would prefer to divert cash to the Pluto project by delaying a second proposed mission to search for life on Jupiter's watery moon Europa, *Science* reports. The White House, on the other hand, is enthralled with the search for extraterrestrial life and so is pushing to go to Europa, reports *Science*. (Perhaps in search of absentee ballots.)

In a related story, the unresolved U.S. presidential election made headlines in both, *Science* and *Nature*. The two magazines note that most important science policy positions will not be appointed until long after the election has been decided. That could leave scientists in the dark for months. *Nature* reports that three issues appear to divide the parties: global warming, embryonic stem-cell research, and national missile defense. *Nature* also reports that the presidential uncertainty has "cast a heavy pall over this week's climate treaty talks in the Hague." Oddly enough, some environmentalists suspect oilman Bush has a better chance of negotiating a treaty than Gore. Why? It's a convoluted argument; just read the *Nature* article.

Split votes and political wrangling also left a mark on the usually pristine world of particle physics this week. Despite tantalizing evidence for the Higgs boson, the last undiscovered particle predicted by the Standard Model of particle physics, CERN decided to close the Large Electron-Positron collider. The decision by Luciano Maiani, director-general of CERN, ends all hope that CERN will find the elusive particle before the new Large Hadron Collider is installed, report both *Science* and *Nature*.
